# Real‐time genomics for One Health

**DOI:** 10.15252/msb.202311686

**Published:** 2023-06-16

**Authors:** Lara Urban, Albert Perlas, Olga Francino, Joan Martí‐Carreras, Brenda A Muga, Jenniffer W Mwangi, Laura Boykin Okalebo, Jo‐Ann L Stanton, Amanda Black, Nick Waipara, Claudia Fontsere, David Eccles, Harika Urel, Tim Reska, Hernán E Morales, Marc Palmada‐Flores, Tomas Marques‐Bonet, Mrinalini Watsa, Zane Libke, Gideon Erkenswick, Cock van Oosterhout

**Affiliations:** ^1^ Helmholtz AI, Helmholtz Zentrum Muenchen Neuherberg Germany; ^2^ Helmholtz Pioneer Campus, Helmholtz Zentrum Muenchen Neuherberg Germany; ^3^ School of Life Sciences, Technical University of Munich Freising Germany; ^4^ Nano1Health SL, Parc de Recerca UAB Campus Universitat Autònoma de Barcelona Barcelona Spain; ^5^ Department of Anatomy University of Otago Dunedin New Zealand; ^6^ Biological Department Machakos University Machakos Kenya; ^7^ BioTeam, Inc. Middleton Massachusetts USA; ^8^ Bioprotection Aotearoa Lincoln University Lincoln New Zealand; ^9^ Plant and Food Research Auckland New Zealand; ^10^ Center for Evolutionary Hologenomics The Globe Institute, University of Copenhagen Copenhagen Denmark; ^11^ Hugh Green Cytometry Centre Malaghan Institute of Medical Research Wellington New Zealand; ^12^ Department of Biology, Ecology Building Lund University Lund Sweden; ^13^ Institute of Evolutionary Biology Universitat Pompeu Fabra‐CSIC, PRBB Barcelona Spain; ^14^ Catalan Institution of Research and Advanced Studies (ICREA) Barcelona Spain; ^15^ CNAG Centre of Genomic Analysis Barcelona Spain; ^16^ Institut Català de Paleontologia Miquel Crusafont Universitat Autònoma de Barcelona Barcelona Spain; ^17^ San Diego Zoo Wildlife Alliance Escondido CA USA; ^18^ Instituto Nacional de Biodiversidad Quito Ecuador; ^19^ Fundación Sumak Kawsay In Situ Cantón Mera Ecuador; ^20^ Field Projects International Escondido CA USA; ^21^ School of Environmental Sciences University of East Anglia Norwich UK

**Keywords:** global health, nanopore sequencing, nature conservation, One Health, real‐time genomics, Chromatin, Transcription & Genomics, Evolution & Ecology

## Abstract

The ongoing degradation of natural systems and other environmental changes has put our society at a crossroad with respect to our future relationship with our planet. While the concept of One Health describes how human health is inextricably linked with environmental health, many of these complex interdependencies are still not well‐understood. Here, we describe how the advent of real‐time genomic analyses can benefit One Health and how it can enable timely, in‐depth ecosystem health assessments. We introduce nanopore sequencing as the only disruptive technology that currently allows for real‐time genomic analyses and that is already being used worldwide to improve the accessibility and versatility of genomic sequencing. We showcase real‐time genomic studies on zoonotic disease, food security, environmental microbiome, emerging pathogens, and their antimicrobial resistances, and on environmental health itself – from genomic resource creation for wildlife conservation to the monitoring of biodiversity, invasive species, and wildlife trafficking. We stress why equitable access to real‐time genomics in the context of One Health will be paramount and discuss related practical, legal, and ethical limitations.

## Graphical abstract

The One Health concept describes the inextricable link between human and environmental health. This review discusses how real‐time genomics can improve our understanding of the interdependencies within ‘One Health’ and enable timely assessments and interventions in many contexts.

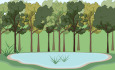

## Introduction

The COVID‐19 pandemic has catapulted the concept of One Health into the center of public attention, showcasing how human health is inextricably linked with the health of our planet (de León *et al*, 2021; van Oosterhout, 2021; One Health High‐Level Expert Panel *et al*, 2022). Although we do not fully understand the connection between the emergence of zoonotic diseases, wild habitat destruction, and biodiversity loss (Keesing & Ostfeld, 2021), projected environmental changes will undoubtedly place an additional burden on planetary and human health. Society is now at a crossroad with respect to our future relationship with planetary health, and our window to act is rapidly closing. This urgency is reflected by the ongoing political, public, and scientific discussions led by the United Nations (UN) Convention on Biological Diversity (CBD), the International Union for Conservation of Nature (IUCN Red List, 2012), the Rockefeller Foundation–Lancet Commission on Planetary Health (Whitmee *et al*, 2015), the Earth Biogenome Project (EBP; Lewin *et al*, 2018), and governing bodies such as the UN Conferences of the Parties (COP) for biodiversity protection (COP15, 2022) and climate changes (COP26, 2021).

**Figure 1:**
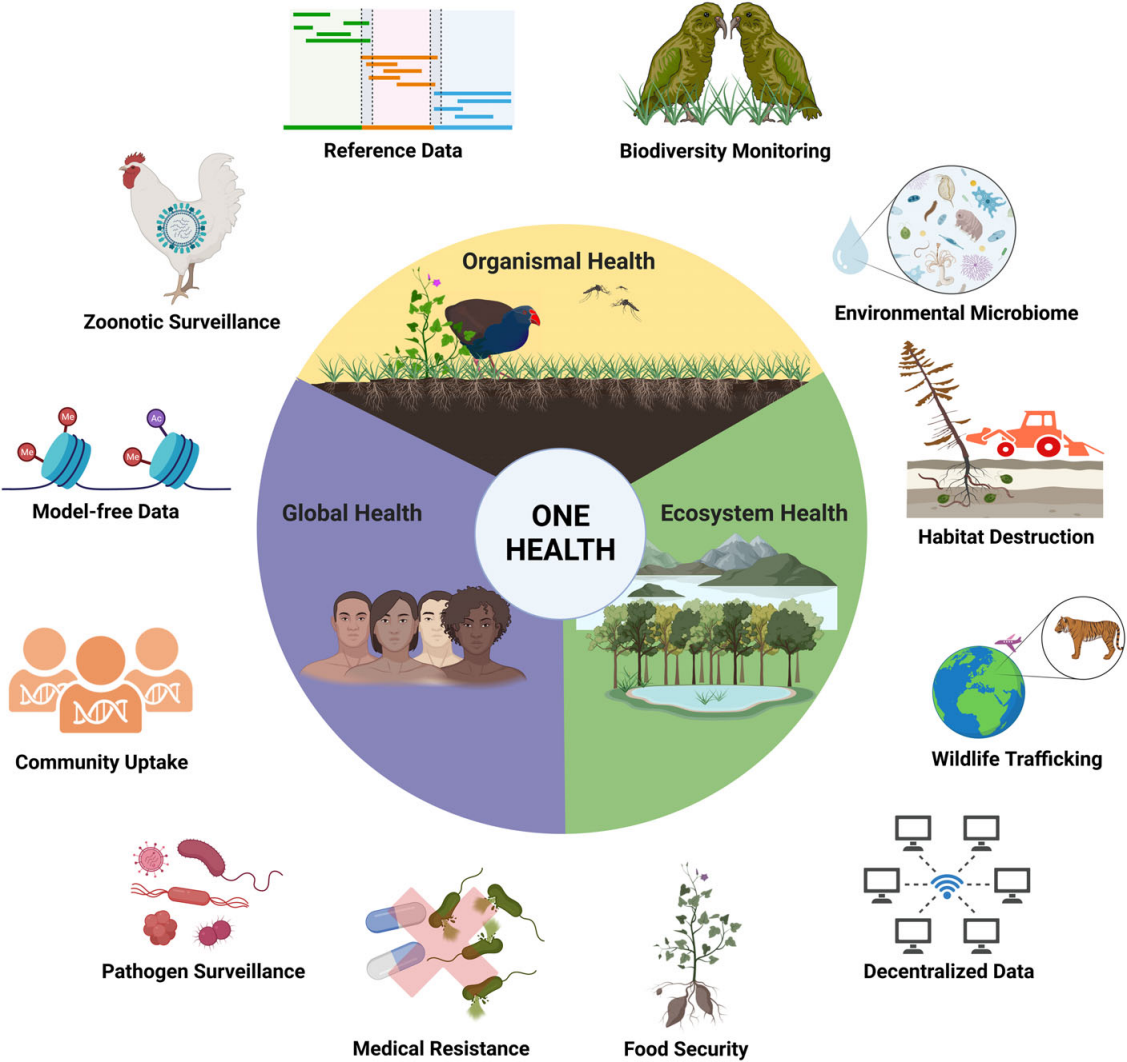
The One Health concept The One Health concept affirms that global human, organismal, and ecosystem health are inextricably linked (*inner circle*). The application of real‐time genomic approaches can help us understand and support One Health at the intersection of these different health concepts (*outer circle*).

Molecular biology can offer practical solutions to environmental challenges, yet it is often discounted by many frontline strategies (Rodríguez‐Martínez *et al*, 2022). Here, we describe how the advent of real‐time genomic analyses can benefit One Health, showing how it enables timely and in‐depth ecosystem health assessments. We discuss how real‐time genomics is becoming instrumental in guiding efficient intervention strategies, presenting examples and highlighting potential future trajectories and limiting factors (Fig 1).

## Real‐time genomics

The need for real‐time genomics has been made clear by recent outbreaks of emerging infectious diseases. Given the exponential growth rates, high transmission potential, and frequent instances of drug resistance of the causative pathogens, it is important to achieve diagnosis turnaround times of hours rather than days or weeks, which precludes the option of transporting samples to large international centers (Gardy & Loman, 2018). In the past two decades, the development of increasingly smaller and cheaper bench‐top sequencing instruments for the first time allowed the use of next‐generation sequencing technologies in local laboratories and clinical settings (Quick *et al*, 2014).

**Figure 2:**
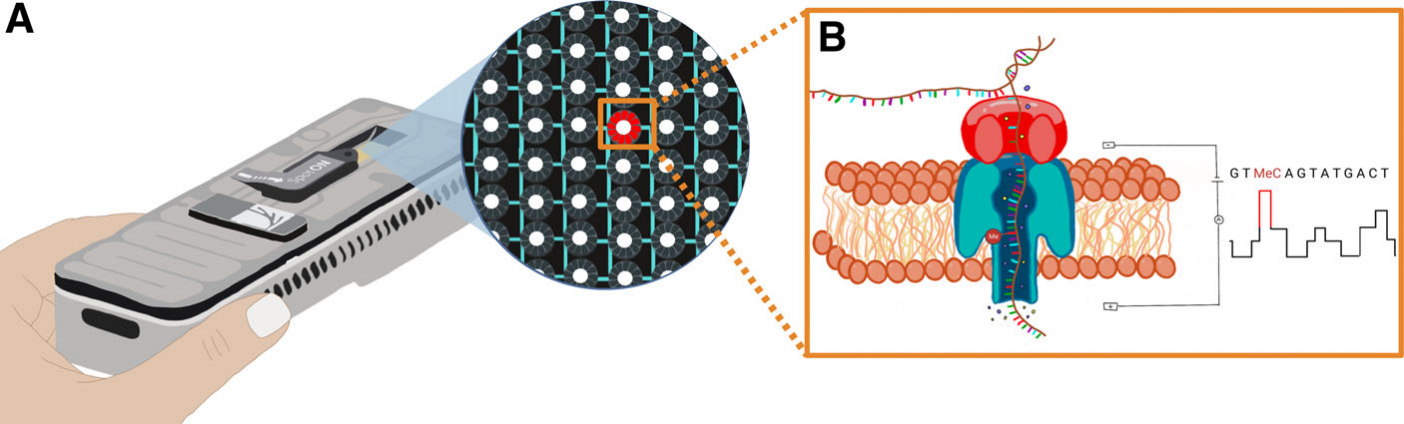
Real‐time nanopore sequencing technology (A) The portable nanopore sequencing device MinION (version Mk1b); the disposable flow cell consists of a fluid‐impermeable polymer membrane with sequencing wells that contain nanopores and that are connected to electrical current sensing circuits to take snapshots of the electrical state of the well at a fixed sampling rate (currently 4 or 5 kHz). (B) An electrical potential across the membrane ensures an ionic flow through the nanopores. When a single‐nucleotide strand passes through the nanopore at a controlled translocation speed, this results in a characteristic disruption of the ionic current that can be basecalled into genomic information such as nucleotide sequence composition and epigenetic modifications. As nucleotides would naturally move through the nanopores too fast for the electrical circuits to detect differences in the ionic current due to individual bases, a helicase is added to the nucleotide strands as an adapter protein. This helicase docks to the nanopores, unwinds the double‐stranded DNA if applicable, and ratchets a single‐nucleotide strand through the nanopore at a controlled translocation speed. While the standard speed has been set to 400 b/s, temperature control can marginally impact translocation speed and, with that, sequencing accuracy. At a sampling rate of 4 kHz, each base is therefore assessed by approximately 10 electrical snapshots.

A new milestone was reached nearly a decade ago when Oxford Nanopore Technologies released its highly portable and cost‐efficient real‐time genomic sequencing device, the MinION (Quick *et al*, 2014; Ip *et al*, 2015; Fig 2A). Nanopores are tiny purpose‐mutated protein pores that enable the sequencing of nucleotides by measuring the disruption of their internal ionic current while DNA and RNA strands pass through them as “squiggle” signal. As specific combinations of nucleotides result in characteristic disruptions of the ionic current, this squiggle signal can be base called rapidly into genomic data using dedicated algorithms such as efficient neural networks (Wick *et al*, 2019; Fig 2B). As such, squiggle signal is model‐free and can incorporate any chemical characteristics of the DNA and RNA strands down to atomic resolution, such as epigenomic modifications. In combination with powerful and parallelizable computers such as graphics processing units (GPUs), this basecalling can happen rapidly, at the speed of sequencing itself. Genomic data can thus both be generated and analyzed in real time and at the point‐of‐care, for example, in the clinical or fieldwork setting (Quick *et al*, 2016). As nanopore sequencing remains the only disruptive technology to date that allows for portable real‐time genomic analyses, it has been leveraged worldwide to break down barriers and improve the accessibility and versatility of genomic sequencing.

### Advantages of real‐time genomics

Nanopore‐based real‐time genomics offers unique advantages. First, real‐time sequencing and basecalling allow for selective sequencing (aka “adaptive sampling”) by rejecting or accepting nucleotide sequences after minimal sequencing effort when the sequence matches a target of interest (Kovaka *et al*, 2021; Payne *et al*, 2021). Such computationally informed enrichment enables researchers to cost‐effectively sequence the genomic data from a specific organism (Urban *et al*, 2023), taxonomic group (Bao *et al*, 2021), or genomic region (Payne *et al*, 2021).

Real‐time sequencing of native DNA strands further enables the retrieval of long sequencing reads including “ultra‐long” reads of several hundred kilobases (kb; Jain *et al*, 2018) and “whales” of several Megabases (Mb) in length (Payne *et al*, 2019). Such long reads enable previously impossible genomic assemblies (e.g., of centromeres and other long tandem repeat arrays) and can improve the quality and contiguity of existing assemblies. They furthermore allow for the phasing of variants and the creation of near‐complete metagenome‐assembled genomes (MAGs) from mixed organismal communities (Jain *et al*, 2018; Nurk *et al*, 2022; Sereika *et al*, 2022).

The native sequencing extends to RNA sequencing, where nanopore sequencing can yield reliable abundance estimates of full‐length transcripts, without introducing biases due to reverse transcription or amplification (Garalde *et al*, 2018). Direct RNA sequencing has, for example, led to the fast discovery of previously undetected viral quasi‐species (Viehweger *et al*, 2019). Finally, the model‐free nature of nanopore squiggle data means that raw data can *a posteriori* be used to retrieve more accurate data and more information than just nucleotide sequence composition, just by training new basecalling algorithms (preprint: Stoiber *et al*, 2017; Wan *et al*, 2022).

## Monitoring environmental health

### Species extinction

The present rate of environmental change is the fastest the Earth has experienced since the last mass extinction approximately 65 million years ago (Mya). Climate change, habitat destruction, pollution, invasive species, overexploitation, and other human‐mediated threats have already resulted in a significantly elevated extinction rate of species which has been recognized as the planet's 6th mass extinction (Barnosky *et al*, 2011; IUCN Red List, 2012). According to the Red List, the population size of 34,432 species (47.6%) is declining, compared to only 1,271 species (1.8%) with increasing population numbers and 36,636 (50.6%) that are stable (IUCN Red List, 2012). The reduction in the effective population size increases genetic drift and the rate of inbreeding, resulting in a loss of genetic diversity. Inbreeding further leads to an increase in the genetic load that becomes expressed (i.e., the realized load; Bertorelle *et al*, 2022), resulting in inbreeding depression. The effects of such genomic erosion can be felt many generations after immediate threats have abated. Even when successful conservation manages to recover the population numbers after a bottleneck, the species may still be at high risk of extinction (Jackson *et al*, 2022). As current policy‐making heavily depends on present and past estimates of species extinction risks (COP15, 2022), real‐time genomic approaches can help rapidly assess the true impact of environmental change on ecosystem composition and functioning by comprehensively describing the genomic erosion of species that could spiral them into an extinction vortex and thereby remedying its potential impacts (preprint: van Oosterhout *et al*, 2022; Theissinger *et al*, 2023).

### Cataloging reference data

Whole‐genome data of threatened species are urgently needed to assess such genomic erosion (preprint: van Oosterhout *et al*, 2022; Theissinger *et al*, 2023). Importantly, existing conservation and extinction assessments are taxonomically biased to well‐studied taxa such as vertebrates (Cowie *et al*, 2022). Ambitious projects such as the EBP intend to catalog all eukaryotic biodiversity and provide a reference for future genomics‐based biodiversity studies (Lewin *et al*, 2018; Ebenezer *et al*, 2022; Formenti *et al*, 2022). While the real‐time component can speed up such efforts, the long and ultra‐long reads produced by nanopore sequencing can greatly facilitate the generation of reference genomic data across the tree of life by providing an anchor for high‐quality, haplotype‐resolved reference genomes. This is particularly relevant to polyploid taxa which represent a significant proportion of eukaryotic life and are particularly difficult to assemble. Moreover, single reference genomes represent a very limited portion of all genomic variation within a species. To better identify structural variation relevant for conservation (Qin *et al*, 2021), it will be necessary to expand from simplified haploid reference genome toward characterizing pan‐genomes (Bayer *et al*, 2020).

The portability of nanopore sequencers hereby enables generating such reference data *in situ*, meaning that, for the first time in the genomic era, the data can be produced close to the species' origin, putting such research in line with CITES (CITES, 1983) and CARE (Global Indigenous Data Alliance, 2022) initiatives as well as data sovereignty principles, as, for example, specified by the Nagoya Protocol on Access and Benefit Sharing (CBD, 2010). The recently launched ORG.one project is an initiative that promotes the uptake of reference genomic data production for endangered animal species. It does this by facilitating the usage of nanopore sequencing for creating high‐quality data with additional support for downstream computational processing and assembly of such data through tailored algorithms and provision of adequate computing power (Oxford Nanopore Technologies, 2022b).

### *In situ* biodiversity monitoring

The portable character of real‐time nanopore sequencing allows for fast *in situ* biodiversity assessments, with the potential to directly impact wildlife conservation decisions in the field (Blanco *et al*, 2020; Pomerantz *et al*, 2022). Nanopore sequencing has already been leveraged for species identification in the field, to generate genomic data for endangered and cryptic species, perform rapid census reports, monitor hybridization zones, and detect the presence of invasive species (Menegon *et al*, 2017; Pomerantz *et al*, 2018; Maestri *et al*, 2019; Blanco *et al*, 2020; Egeter *et al*, 2022; Urban *et al*, 2023). These applications have proven especially valuable when it comes to direct conservation management adjustments in remote environments, where sample storage and transport would be prohibitive for any genomic study (Krehenwinkel *et al*, 2019; Watsa *et al*, 2020), and in countries where access to laboratory facilities and conventional genomic sequencing approaches remains challenging (Hetu *et al*, 2019). This holds the promise of having local conservationists and communities of indigenous people monitor and manage the biodiversity of the ecosystems they live in, and of supporting the democratization of molecular analyses through local research and teaching (Blanco *et al*, 2020; Watsa *et al*, 2020) as well as data and benefit sharing (Mc Cartney *et al*, 2022) (Box 1).

Box 1 **The *In Situ* Lab Initiative**

**Figure 3:**
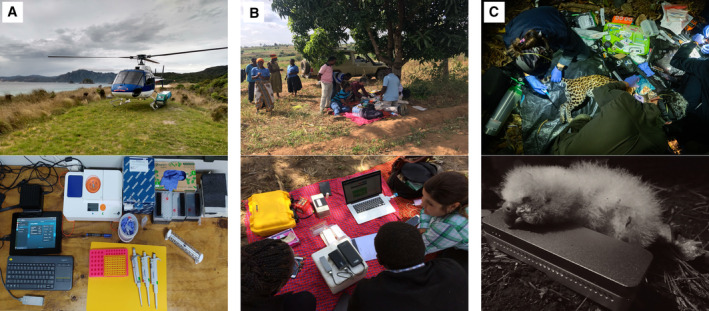
Applications of real‐time genomics for One Health at the point‐of‐care (A) *In situ* applications on remote islands in Aotearoa New Zealand through the employment of portable laboratory, sequencing, and computational equipment (photo credit: Kākāpō Recovery Team, Department of Conservation, New Zealand). (B) Food security application and community involvement on subsistence farms in Tanzania to rapidly diagnose African cassava mosaic viruses in cassava plants (photo credit: Laura Boykin Okalebo; Jo‐Ann L Stanton). (C) Biodiversity monitoring applications for the conservation of critically endangered wildlife in Ecuador (*top*) and Aotearoa New Zealand (*bottom*) (photo credits: The In Situ Laboratory Initiative; Kākāpō Recovery Team).

The *In Situ* Lab Initiative (ISL) was established in 2020 as a complementary model to present‐day global One Health programs for emerging disease detection in humans that are implemented in a centralized, top–down fashion. The ISL aims to empower local stakeholders such as universities, zoos, conservation non‐governmental organizations, and governments to update their wildlife or environmental surveillance efforts with modern, low‐cost, and portable molecular toolkits to engage in One Health projects in ways that are meaningful to their constituents – which is not necessarily in accordance with the goals of the international community. The ISL facilitates efforts to establish decentralized wildlife surveillance labs worldwide by fostering a network of labs that curate, standardize, and share protocols. Participants of the ISL agree to maintain certain standards of biosafety, data management, protocol sharing, and project management. Over time, as the diffuse partner network expands, and projects in one location overlap with those in another, collaboration results in convergence on best practices. A versatile and competent network of locally run laboratories can redirect resources and respond effectively to gather information on emergent diseases or ecological threats. With functioning labs in Peru, Ecuador (Fig 3C), and soon Indonesia, Vietnam, and Rwanda, the ISL aligns with the Nagoya Protocol by avoiding the exportation of genetic samples and focusing on benefit sharing within communities by centering their participation in community‐driven laboratory‐based investigations. Central to this effort is nanopore sequencing, which allows for all genetic research to be carried out at the site of sample origin, creating positive conservation incentives for genetic resources.

Long nanopore sequencing reads further assist such biodiversity assessments by allowing targeting full‐length marker genes in metabarcoding studies (Krehenwinkel *et al*, 2019) or complete mitochondrial genomes (Malukiewicz *et al*, 2021), which can increase the taxonomic resolution of genetic studies. Thanks to the advent of nanopore selective sequencing based on digital sequence information, it has become feasible to enrich environmental genomic material extracted from water, soil, or fecal samples for gene region‐ or species‐specific targets, allowing for non‐invasive genomic biodiversity monitoring (Wanner *et al*, 2021). Given that long reads contain several genetic variants, they can not only distinguish between species but also reliably perform individual identification based on haplotypes of several kb in length. This has been shown to work for soil environmental DNA monitoring of the critically endangered kākāpō (*Strigops habroptilus*) in Aotear New Zealand (Fig 3A; Urban *et al*, 2023), with the potential promise of extending non‐invasive biodiversity monitoring to within‐species assessments of genetic diversity and genomic erosion.

### Combating wildlife trafficking

International wildlife trafficking is one of the largest organized transnational crimes, involving the smuggling, poaching, capture, or collection of protected species (Smart *et al*, 2021). Besides representing one of the major threats to biodiversity, it also entails a biosecurity risk because the unregulated trade of animals can mediate disease transmission (Rush *et al*, 2021). While local and international laws and regulations prosecute wildlife trafficking (CITES, 1983), actual prosecution of wildlife crime is very often impaired due to improper identification of the species or population, and as a result the country or area of origin after confiscation (Gouda *et al*, 2020).

The *in situ* application of real‐time nanopore sequencing to wildlife trafficking opens new avenues for direct application to confiscated samples at borders and airports. Traditional genetic methods such as the sequencing of targeted marker genes or of mitochondrial DNA have become part of the daily toolkit of wildlife forensics (Wasser *et al*, 2015; Smart *et al*, 2021). Portable sequencing technologies that produce long reads can further increase the taxonomic resolution and accuracy when determining the place of origin of the confiscated sample. This has previously been demonstrated through *ex situ* next‐generation sequencing of confiscated live chimpanzees (Fontsere *et al*, 2022). Here, long‐read sequencing can potentially be applied for reintroducing such confiscated individuals to their populations of origin, as well as for detecting poaching hotspots so that authorities can enforce laws in order to protect wildlife. The same technology has been used to help identify CITES‐listed shark species on a food market in India through genome skimming (Johri *et al*, 2019). Such *in situ* monitoring at locations of high human and trade traffic can be extended to monitor the spread of invasive species and infectious disease, which might be associated with any wildlife material.

### Application of model‐free sequencing

The model‐free nature of nanopore sequencing means that any modification of the genomic or transcriptomic material can be detected – as long as a basecalling algorithm can be trained to detect such a modification beyond the variation in the sequence composition (preprint: Stoiber *et al*, 2017; Wan *et al*, 2022). This has been leveraged to call epigenetic modifications such as DNA methylation and histone modifications (Simpson *et al*, 2017; preprint: Stoiber *et al*, 2017; Yue *et al*, 2022), which provide another rich level of information for assessing biodiversity and its function. Epigenetic variation represents an important part of biodiversity and can provide information about the ecological or environmental components of species and populations (Moore *et al*, 2013; Lacal & Ventura, 2018). Such modifications can influence an offspring's attributes and fitness conditions, potentially impacting several generations (Bošković & Rando, 2018) and further contributing to a potential extinction vortex. Epigenetic changes can further be used to identify species‐ and population‐specific adaptations to changing environments such as climate change (Lighten *et al*, 2016), or exposure to toxic substances (Fernández *et al*, 2014). Nanopore sequencing has already been used to study the epigenomic landscape of medaka fish (Leger *et al*, 2022) and to evaluate functionally important phenotypes in bacteria (Beaulaurier *et al*, 2019).

Model‐free sequencing of native genomic material has the potential to *de novo* discover any modification of the nucleotide strand down to the atomic level (preprint: Stoiber *et al*, 2017). This has, for example, been leveraged to distinguish bacterial from human DNA on the squiggle signal level, allowing for efficient enrichment of microbial genomic information through nanopore selective sequencing (Bao *et al*, 2021). In such cases, the analysis of the basecalling algorithms through explainable machine learning (ML) approaches can teach us more about hidden molecular differences between taxonomic groups. We envision that the usage of squiggle data for functional assessments of genomic data will open up new possibilities for epigenetic and comparative genomic research.

## Environmental, organismal, and human health

### The bidirectional relationship between environmental and human health

In contrast to several organisms that face an elevated extinction risk, many pathogenic organisms such as fungi, bacteria, and viruses are thriving as a result of the rapidly changing environmental conditions. The extremely high biomass and population densities of our livestock and crops (Bar‐On *et al*, 2018) in combination with their relatively low levels of genetic diversity (Zhang *et al*, 2018) make them susceptible to emerging infectious diseases (van Oosterhout, 2021). Habitat destruction and wildlife trade further increase the risk of pathogen spillover events from wildlife to livestock (Rush *et al*, 2021). In turn, close contacts between humans and our livestock promote the evolution of zoonotic disease. Hybridization between previously isolated pathogens allows for genetic introgression, potentially resulting in hybrid speciation in pathogens of humans (Tichkule *et al*, 2022), animals (Borlase *et al*, 2021), and plants (preprint: Mathers *et al*, 2022; Rogério *et al*, 2022). All these developments are shifting the dynamic co‐evolutionary equilibria in favor of pathogens (van Oosterhout, 2021), putting severe pressure on our society and environment, both now and in the future.

Given the global scale and complex nature of the interdependencies between environmental, organismal, and human health, real‐time genomics has the potential to provide fast diagnostic tools at the point‐of‐care anywhere in the world. Nanopore sequencing has been adopted by a wide variety of stakeholders, including environmental scientists, conservationists, genetic engineers, and health practitioners, to save valuable time and implement appropriate control measures – in the clinical, veterinarian, agricultural, environmental, biodiversity monitoring, or wildlife health setting (Quick *et al*, 2016, 2017; Vanmechelen *et al*, 2017; Theuns *et al*, 2018; Kafetzopoulou *et al*, 2019; Freed *et al*, 2020; Rambo‐Martin *et al*, 2020; Street *et al*, 2020; Charalampous *et al*, 2021; Vandenbogaert *et al*, 2022).

### Zoonotic disease

Zoonoses are infectious diseases caused by host switching of pathogenic viruses, bacteria, fungi, or protists (Jones *et al*, 2008). The spillover of a zoonotic virus from bats to humans via a still unknown animal species has been suggested as the potential source of the recent COVID‐19 pandemic caused by the SARS‐CoV‐2 virus (Yoo & Yoo, 2020; Temmam *et al*, 2022), showcasing the potential impact of zoonoses on a global scale. In this context, real‐time genomics can tackle zoonoses in remote areas (Gardy & Loman, 2018), while long sequencing reads can help identify novel genomic variants and distinguish genuine recombinants from chimeras, i.e., sequencing or assembly artifacts that can be generated when analyzing mixed infections.

Shortly after the first release of nanopore sequencing technology, an *in situ* real‐time genomic surveillance program was established to track the Ebola virus epidemic in West Africa (Quick *et al*, 2016). It was further used to track the Zika virus epidemic in Brazil (Faria *et al*, 2016; Quick *et al*, 2017), the COVID‐19 pandemic (Fauver *et al*, 2020; Freed *et al*, 2020; Meredith *et al*, 2020), and several other pathogens, including viruses causing Lassa and yellow fever, avian influenza, and rabies (Kafetzopoulou *et al*, 2019; Brunker *et al*, 2020; Hill *et al*, 2020; Rambo‐Martin *et al*, 2020; Crossley *et al*, 2021). The rapid operability has also made nanopore technology the first go‐to tool in the multi‐country monkeypox outbreak in 2022, resulting in the first draft genome of the virus shortly after the beginning of the outbreak (Isidore *et al*, 2022). Real‐time genomics can help inform medical responses, vaccine development, and public health management by providing a better understanding of transmission routes and frequency. These whole‐genome assessments can detect various pathogenic species across diverse taxonomic groups through metagenomic approaches, which can simultaneously identify and characterize diverse microorganisms (e.g., viral, bacterial, or fungal) with precision and even detect yet‐to‐emerge pathogens (Ko *et al*, 2022).

*In situ* real‐time genomics can generate genomic data of pathogens in a decentralized manner, improving the surveillance in previously neglected geographic regions and low‐income countries. Existing large‐scale viral monitoring projects such as Prezode (Peyre *et al*, 2021), the Global Virome Project (Carroll *et al*, 2018), or Virion (Carlson *et al*, 2022) have already made use of publicly available big data to detect novel potentially pathogenic viruses or viral variants (Carroll *et al*, 2018; Albery *et al*, 2021). Nanopore sequencing with its potential for automatization and decentralized deployment provides a unique opportunity to further augment and federate worldwide data and use it to enable predictions of novel threats to human health through ML applications (Carlson, 2020). We envision that such decentralized data production in combination with efficient ML can help enable instant, globalized communication about public health risks.

### Food security

The challenge of securing food supply for human society is growing in both size and inequity and is directly linked to many other One Health‐related problems. Real‐time genomics can improve global food security and contribute to economic stability, for example, by reducing crop loss through diagnosing plant diseases and pests accurately and early (Boykin *et al*, 2018). The creation of global genomic reference data can further inform and accelerate global food production, ultimately democratizing the benefits of genomic research. For example, nanopore sequencing has revolutionized genome sequencing of important plant species such as crops, enabling the accurate assembly of their often large and highly repetitive genomes (Ibe, 2022). Nanopore sequencing has also shown a link between antimicrobial resistance and bacterial virulence in livestock and their human farmers (Viñes *et al*, 2021), highlighting the role of livestock as a reservoir of pathotypes with zoonotic potential and as a potential source of food insecurity.

The potential impact of diagnostic sequencing in real time was convincingly demonstrated in its application to the cassava plant in Tanzania in 2017 and 2018, which feeds 800 million people worldwide (Fig 3B; Boykin *et al*, 2019). During a visit to a subsistence farm run by a women's chama (Swahili for collective), portable DNA extraction and nanopore sequencing were used to identify particular strains of African cassava mosaic virus in cassava plants. Rapid diagnosis allowed replacing the crop with two cassava varieties tolerant to the identified viral strains in time for the 2018 harvest. This ensured food security as the virus‐tolerant cassava varieties produced around 35 tons per hectare for sale, whereas previous crops from the chama had not yielded enough harvest for the market. When considering the average market value of cassava in 2018, the associated production costs, and the household incomes in Tanzania (National Bureau of Statistics, 2019), this real‐time genomics‐informed intervention provided the chama with surplus income equivalent to approximately 3.7 times the average monthly income in Tanzania for 2018 (also see “Extant Challenges”).

### The environmental microbiome

Real‐time metagenomics surveillance approaches can also be used to describe the natural environmental microbiome, for example, from non‐invasive samples such as air, water, or soil, and help us better understand the functional interactions between humans, the environmental microbiome, and ecological change (Gowers *et al*, 2019; Haan & Drown, 2021; Edwards *et al*, 2022). Increased anthropogenic pressures and rapid climate change can also leave their footprint on these microbial communities. For example, antimicrobials such as antibiotics, antifungals, and disinfectants have been overused in the clinical and agricultural setting, leading to resistances and environmental pollution. When coupled with extreme weather patterns and higher temperatures, it can lead to the spread of superbugs, i.e., microorganisms that are resistant to most medications (UNEP, 2023).

Real‐time environmental metagenomics has the potential to unmask such complex relationships between human and environmental health in the context of One Health. Known and novel pathogens and their transmission dynamics have been identified from freshwater or wastewater sources to describe potential human health consequences (Izquierdo‐Lara *et al*, 2021; Urban *et al*, 2021), and harmful algal blooms that can directly influence ecosystem services have been detected early (Hatfield *et al*, 2020; preprint: Koeppel *et al*, 2022). Beyond taxonomic assignments of microorganisms, the long reads of nanopore sequencing have been used to detect medical resistance‐ and virulence‐associated genes and gene clusters, allowing for direct functional predictions and transmission surveillance – for example for drug resistance profiling of tuberculosis‐causing *Mycobacterium* (Chan *et al*, 2020), malaria‐causing *Plasmodium falciparum* (Runtuwene *et al*, 2018), Leishmaniasis‐causing *Leishmania infantum* (Martí‐Carreras *et al*, 2022), and a wide range of other taxa (Ashton *et al*, 2015; Břinda *et al*, 2020; Bokma *et al*, 2021).

## A vision for equitable and inclusive One Health

### Accessibility

Real‐time genomics has the potential to increase equity and inclusion with respect to access to One Health research through its disruptive and distributed nature. Nanopore sequencing has been developed with the aim of being accessible to “anyone, anywhere”, which has been achieved through reduced upfront investment costs and by pairing it with portable DNA and RNA extraction and data analysis approaches (Palatnick *et al*, 2020; Oxford Nanopore Technologies, 2022b). This has had important implications for shifting the current hierarchical genomic framework with high‐volume large sequencing centers to highly distributed low‐volume bespoke sequencing. This solves logistically difficult storage and transport of samples, reduces the risk of invasive pathogens being transported along with the samples, and circumvents issues related to permits and travel restrictions.

### Community

The *in situ* application of real‐time genomics for One Health enables its uptake by communities themselves, reducing the harmful practices of neocolonialism and helicopter science (Adame, 2021; Haelewaters *et al*, 2021), with the potential to democratize and diversify scientific practice (Nagaraj *et al*, 2020). If used in the right way, *in situ* applications can support indigenous rights – such as demonstrated by the concept of “ahi kā” or “keeping the home fires burning” by Māori communities in Aotearoa New Zealand. This means that genomic material and data can remain in the hands of the involved communities, who can subsequently be in control of their own diagnostics and maintain self‐determination.

Māori, like many other indigenous communities globally, are a recognized force in the front lines of biosecurity surveillance and conservation management (Lambert & Mark‐Shadbolt, 2021). In Te Ao Māori (the world of the Mori), the entire Earth is known as Papatūānuku, the Earth mother, and all life depends upon Papatūānuku for their wellbeing. People have the option of caring for her to maintain their own health or abandoning her to concentrate on their own short‐term needs. By always keeping in mind the needs of Papatūānuku and the requirements of her immediate whānau, Māori have for a long time been advocates of the importance of One Health, while these interdependencies between environmental and human health have been gradually de‐emphasized by many other cultures.

However, Māori continue to have limited access to the latest technologies in disease diagnostics (Palmer *et al*, 2020), often due to financial and technical limitations. Efforts to protect their culturally significant species, the environments they exist in, and customary harvesting practices require more accessible tools and training opportunities so that indigenous communities can contribute to, and benefit from, a better, more inclusive biosecurity and conservation system. Emerging pests and pathogens are of great concern both economically as well as presenting a conservation threat to already endangered species. One example is the plant pathogen *Phytophthora agathidicida*, which is the accepted causal agent of kauri (*Agathis australis*) forest diebacks (Weir *et al*, 2015), but which is not well‐understood with respect to transmission and possible prevention. As many of the infected and vulnerable kauri forests are managed by Māori, portable real‐time genomics can provide an accessible and accurate diagnostic tool for the effective identification of emerging pests and pathogens of economic and cultural importance.

### Extant challenges

While real‐time portable genomics through nanopore sequencing has led to increased accessibility, decreased initial financial investment, and offers many opportunities for improving our global understanding of One Health, nanopore sequencing still faces substantial financial, technological, and ethical limitations.

Nanopore sequencing does not require immense upfront investment costs when the freely available portable sequencing devices are being used, but the regular consumable costs can be substantial, especially for low‐ and middle‐income countries. For portable applications, the lowest‐capacity sequencing flow cell is called a Flongle, which costs about US$ 7 each, has an output of hundreds of Mb, and does not have any specific storage requirements. The MinION flow cell costs about US$ 900, has a typical yield of about 5–15 Gb, and has to be stored at fridge temperature. For high‐throughput sequencing devices, the P2 Solo (US$ 10,455 + 1,000 USD per year) is the most affordable, with additional costs of US$ 1,400 per PromethION flow cell resulting in a yield of 50–150 Gb. While optimization of these costs through multiplexing, re‐usage of flow cells, and optimization of DNA extraction and sequencing protocols can result in reasonable costs per base (Blanco *et al*, 2020), pilot studies usually have to be conducted to understand the sequencing throughput and the necessary sequencing depth for each new sample type. This creates uncertainty for the user with respect to financial considerations and data storage. Simultaneously, many countries do not yet have access to reliable suppliers of nanopore sequencing material, resulting in often substantially increased material and shipping costs, or a complete lack of accessibility.

While these financial limitations will have to be resolved in the future, the advantage of real‐time *in situ* application has to be taken into account for any economic considerations. For example, if we consider sequencing costs in the African cassava mosaic virus study described earlier (see “Food security”), we estimated processing costs of US$ 42 per sample. These costs were about a tenth the cost of Illumina sequencing in Tanzania (quoted at US$ 386 in 2018), and the additional turn‐around time of Illumina sequencing at an offshore site would have prevented the replanting of different cassava varieties and therefore any immediate economic and societal benefits for the chama. In other words, where the costs can be met, the benefit of *in situ* sequencing is substantial.

Many practical challenges prevent the widespread and large‐scale uptake of real‐time genomic technology. The lack of stable electrical supply can negatively affect the storage of temperature‐sensitive reagents, which poses a big limitation on long‐term field studies in remote areas (Pomerantz *et al*, 2018; Blanco *et al*, 2020). A lack of internet connection and large amounts of data that accumulate over long periods of time can be limiting factors for the subsequent bioinformatic analysis of sequencing data (Blanco *et al*, 2020). Another important technological limitation of nanopore sequencing has been the inflated sequencing read error rate of up to 8% (Urban *et al*, 2021), which mainly stemmed from difficulties of the nanopores to accurately distinguish homopolymers (Delahaye & Nicolas, 2021) with potential implications for faulty assemblies, false‐positive variant calling, and frameshift errors. Thanks to the latest Kit 14 nanopore sequencing chemistry (introduced in 2022) together with duplex sequencing (basecalling the forward and reverse‐complementary DNA strands in tandem), this sequencing read error rate has decreased to 0.6% (Oxford Nanopore Technologies, 2022a). This means that it is now possible to generate highly accurate and complete assemblies without the need for short‐read polishing (Sereika *et al*, 2022). This high sequencing accuracy is very promising for any future application of nanopore sequencing in the space of One Health.

Community uptake and empowerment to routinely use real‐time genomic‐based diagnostics remain difficult. Access to real‐time genomic technology remains hampered by a lack of automatization, both on the laboratory and on the computational level. Therefore, the actual application of nanopore sequencing still requires advanced molecular biology and analytical skills. Even the application of standard bioinformatic pipelines implemented in Oxford Nanopore Technologies' EPI2ME (EPI2ME Labs, 2023) still requires knowledge about the underlying analysis pipeline and databases for comprehensive interpretation. If this, however, leads to the application of a “lab‐in‐a‐suitcase” without appropriate community engagement and involvement, it could threaten the idea of self‐determined independent applications by local communities and researchers and further perpetuate helicopter research (Haelewaters *et al*, 2021). The difficulty of analyzing and interpreting nanopore data has, for example, been highlighted after in‐field sequencing in Africa (Boykin *et al*, 2019). Subsequent courses organized by the African BecA‐ILRI hub as a 3‐month hybrid training program for African scientists allowed them to learn the basic applications of nanopore sequencing and to simultaneously apply this real‐time genomic technology to study the genetic potential of crops and livestock in the context of food security (BecA‐ILRI hub, 2023). Such training needs to be standardized and made available worldwide (at community‐affordable costs) to enable truly global and equitable access to the advances of real‐time genomic research for One Health.

## Conclusion

We envision that the use of real‐time genomic technologies and their application at the point‐of‐care can improve our understanding of complex interdependencies within the One Health concept, directly informing management decisions *in situ* in clinical, veterinarian, agricultural, conservation, and environmental applications. A paradigm shift is required to really ensure an equitable and global uptake of real‐time genomics. To support decentralized capacities, this will have to involve tackling global disadvantages and rendering the technology flexible with respect to commercialization, and compatible with different cultural models and infrastructure. To increase the accessibility of the technology, further development and automation are required, which would enable its application at airports, in hospitals, at remote wildlife camps, in local communities, or in daily conservation and biosecurity surveillance. The global and decentralized genomic data created this way hold the promise of widening our horizon to implications of the One Health concept that have so far been hidden from us, and would enable us to further adapt our attitude towards the interdependencies between human and environmental health.

## Author contributions

**Lara Urban:** Conceptualization; supervision; visualization; writing – original draft; project administration; writing – review and editing. **Albert Perlas:** Conceptualization; writing – original draft. **Olga Francino:** Conceptualization; writing – original draft. **Joan Martí‐Carreras:** Conceptualization; writing – original draft. **Brenda A Muga:** Conceptualization; writing – original draft. **Jenniffer W Mwangi:** Conceptualization; writing – original draft. **Laura Boykin Okalebo:** Conceptualization; writing – original draft. **Jo‐Ann L Stanton:** Conceptualization; writing – original draft. **Amanda Black:** Conceptualization; writing – original draft. **Nick Waipara:** Conceptualization; writing – original draft. **Claudia Fontsere:** Conceptualization; writing – original draft. **David Eccles:** Conceptualization; visualization; writing – original draft. **Harika Urel:** Visualization; writing – original draft. **Tim Reska:** Visualization. **Hernán E Morales:** Conceptualization; writing – original draft. **Marc Palmada‐Flores:** Conceptualization; writing – original draft. **Tomas Marques‐Bonet:** Conceptualization; writing – original draft. **Mrinalini Watsa:** Writing – original draft. **Zane Libke:** Writing – original draft. **Gideon Erkenswick:** Writing – original draft. **Cock van Oosterhout:** Conceptualization; supervision; writing – original draft.

## Disclosure and competing interests statement

The authors declare that they have no conflict of interest.
